# Sbp1 modulates the translation of Pab1 mRNA in a poly(A)- and RGG-dependent manner

**DOI:** 10.1261/rna.062547.117

**Published:** 2018-01

**Authors:** Alberto Brandariz-Núñez, Fuxing Zeng, Quan Ngoc Lam, Hong Jin

**Affiliations:** 1 Department of Biochemistry, University of Illinois at Urbana-Champaign, Champaign, Illinois 61801, USA; 2 Center for Biophysics and Quantitative Biology, University of Illinois at Urbana-Champaign, Champaign, Illinois 61801, USA

**Keywords:** translational regulation, RNA-binding protein, Sbp1, 5′UTR and polyadenosine-binding protein Pab1

## Abstract

RNA-binding protein Sbp1 facilitates the decapping pathway in mRNA metabolism and inhibits global mRNA translation by an unclear mechanism. Here we report molecular interactions responsible for Sbp1-mediated translation inhibition of mRNA encoding the polyadenosine-binding protein (Pab1), an essential translation factor that stimulates mRNA translation and inhibits mRNA decapping in eukaryotic cells. We demonstrate that the two distal RRMs of Sbp1 bind to the poly(A) sequence in the 5′UTR of the Pab1 mRNA specifically and cooperatively while the central RGG domain of the protein interacts directly with Pab1. Furthermore, methylation of arginines in the RGG domain abolishes the protein–protein interaction and the inhibitory effect of Sbp1 on translation initiation of Pab1 mRNA. Based on these results, the underlying mechanism for Sbp1-specific translational regulation is proposed. The functional differences of Sbp1 and RGG repeats alone on transcript-specific translation were observed, and a comparison of the results suggests the importance of remodeling the 5′UTR by RNA-binding proteins in mRNA translation.

## INTRODUCTION

Translation is responsible for the production of proteins that are essential for cellular functions (Gebauer and Hentze 2004; Sonenberg and Hinnebusch 2007; Spirin 2009). One key feature of translation is that cellular mRNAs are translated differently, as a result of the difference in interactions between mRNAs and the translational machinery (Vogel et al. 2010; Gingold and Pilpel 2011). In principle, for a multistep cellular process like translation, regulation can take place at each step and can target numerous proteins or RNAs involved (Merrick and Hershey 1996). Nevertheless, this process is predominantly regulated during initiation (Sonenberg and Hinnebusch 2009; Jackson et al. 2010; Hinnebusch and Lorsch 2012), the step when interactions between 5′ untranslated regions (5′UTRs) of mRNAs and cellular proteins determine how ribosomes will be recruited to the mRNAs to start translation.

5′UTRs of mRNAs regulate translation via a diverse set of mechanisms (Sonenberg and Hinnebusch 2009; Jackson et al. 2010; Ingolia et al. 2011; Brar et al. 2012; Hinnebusch and Lorsch 2012; Archer et al. 2016; Hinnebusch et al. 2016). Most notably, the 5′UTR of an mRNA often harbors binding sites for regulatory proteins and translational factors. These RNA-binding proteins (RBPs) are versatile not only in their ways of recognizing partner RNAs but also in interacting with other proteins, which enables them broad yet unique functions in regulating post-transcriptional gene expression (Keene and Tenenbaum 2002; Hogan et al. 2008).

Sbp1 is an RNA-binding protein that was initially discovered to coimmunoprecipitate with small nucleolar RNA 10 and 11 (snoRNA10 and snoRNA11) in yeast (Jong and Campbell 1986; Jong et al. 1987; Clark et al. 1990). In addition to its localization in the nucleolus (Jong et al. 1987; Clark et al. 1990), Sbp1 was shown to localize in processing bodies (P bodies) under the stress of glucose starvation in yeast (Segal et al. 2006). Overexpression of Sbp1 results in global translational repression and an increase in the size as well as number of P bodies (Segal et al. 2006). In the same study, Sbp1 was shown to promote decapping of a reporter mRNA with another protein, Dhh1.

In line with functional roles of this protein in translation, overexpression of Sbp1 rescues the defect in nonsense suppression caused by SUP35 mutant (Zadorsky et al. 2015). Furthermore, in a study on RNA-binding protein FUS/TLS-dependent cytotoxicity that causes a subset of familial amyotrophic lateral sclerosis (fALS) using yeast as a model system, overexpression of Sbp1 rescues the toxicity caused by FUS/TLS mutations (Ju et al. 2011).

**FIGURE 1.**
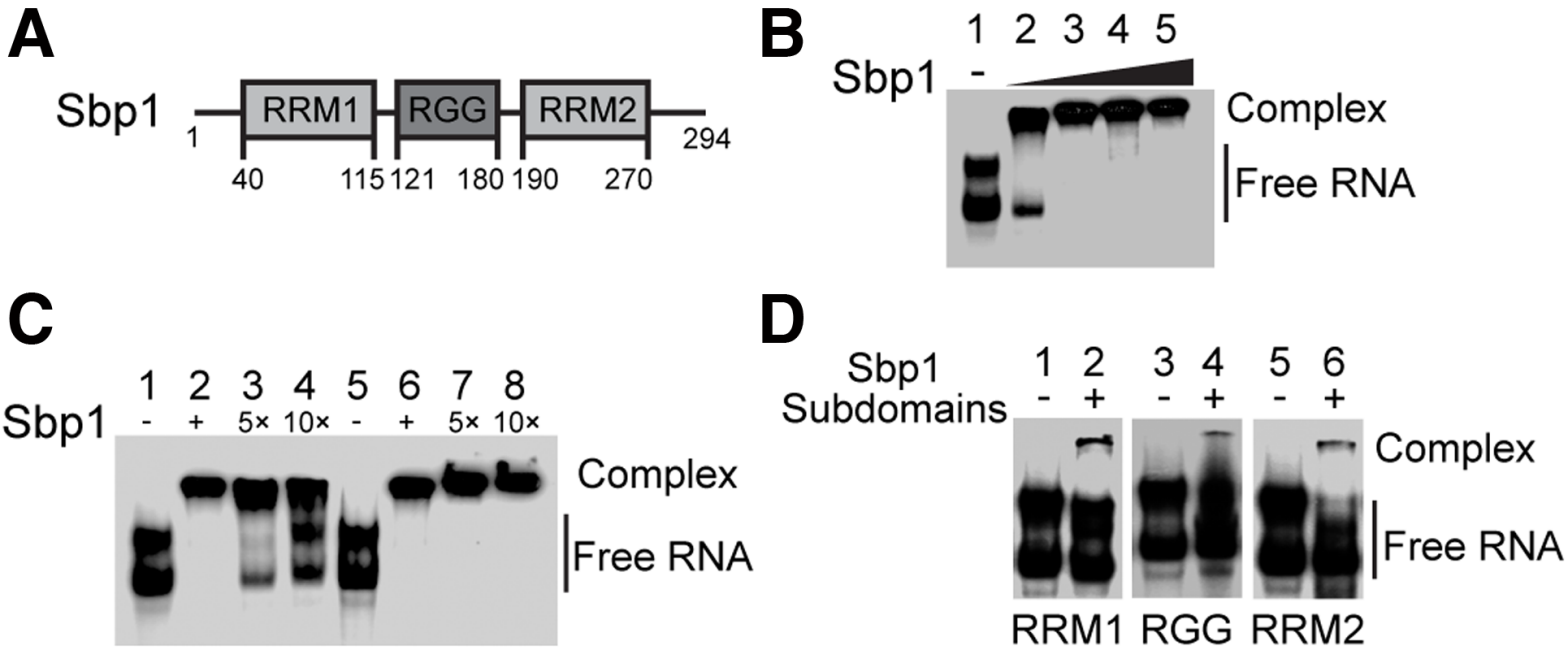
Sbp1 binds to 5′UTR_Pab1_ specifically and cooperatively. (*A*) Schematic representation of Sbp1's domain organization. Amino acid residue numbers at the boundaries of individual domains are indicated. (*B*) An EMSA showing the formation of 5′UTR_Pab1_•Sbp1 complex at 1:1 stoichiometry. 5′UTR_Pab1_ was incubated with increasing concentration of purified Sbp1 (1:0, 1:5, 1:10, 1:15, and 1:20 of 5′UTR_Pab1_:Sbp1 for lanes *1*–*5*, respectively). (*C*) Competition EMSA showing specific interactions between 5′UTR_Pab1_ and Sbp1. Sbp1 was first incubated with ^32^P-5′UTR_Pab1_ (lanes *2* and *6*). An excess of unlabeled 5′UTR_Pab1_ by five- and 10-fold (lanes *3* and *4*) and its complementary RNA, 5′UTR_Pab1-complement_, (lanes *7* and *8*) were used to compete with ^32^P-5′UTR_Pab1_ bound to Sbp1. (*D*) An EMSA showing weak interactions between the 5′UTR_Pab1_ and individual domains of Sbp1. Sbp1_RRM1_ (lane *2*), Sbp1_RGG_ (lane *4*), and Sbp1_RRM2_ (lane *6*) were incubated with 5′UTR_Pab1_. A molar ratio of 1:20 (5′UTR_Pab1_:Sbp1 domain mutants) was used in the experiment. 5′UTR_Pab1_ alone was shown in lanes *1*, *3*, and *5*.

Sbp1 has a domain organization with an N-terminal RNA recognition motif (RRM) domain, a central arginine–glycine –glycine (RGG) domain and a second C-terminal RRM domain (Fig. 1A). Because of the diverse roles that RRM and RGG domains play in mediating RNA–protein, protein–protein, and less frequent but important, RNA–RNA interactions (Cuchalová et al. 2010; Rajyaguru and Parker 2012; Daubner et al. 2013; Thandapani et al. 2013), Sbp1 has the potential to target different mRNAs and regulate their translation in a transcript-specific way. Furthermore, unlike the majority of RGG-motif containing proteins which have their RGG repeats in either N or C terminus (data not shown), the location of RGG repeats between the two RRMs suggests potential function of structural modulation of the RNA target by this protein. Despite the importance, very little is known about molecular mechanisms by which Sbp1 regulates transcript-specific mRNA translation.

Here, we report that Sbp1 inhibits translation of the mRNA that encodes polyadenosine [poly(A)]-binding protein Pab1 in an RNA- and RGG-dependent manner in vitro. We further elucidate the molecular interactions that are important for the translational regulation. Pab1 is an essential regulator of mRNA metabolism in eukaryotes, including splicing (Jenal et al. 2012), 3′ end processing (Jenal et al. 2012), and mRNA export (Bresson and Conrad 2013), localization (Vazquez-Pianzola et al. 2011), translation (Munroe and Jacobson 1990; Tarun and Sachs 1996; Tarun et al. 1997; Wells et al. 1998), and degradation (Bresson and Conrad 2013). Pab1 not only binds to the 3′ poly(A) tail and other adenosine-rich (A-rich) regions of cellular mRNAs (Gilbert et al. 2007), but also directly interacts with the eukaryotic mRNA cap-binding complex via binding with its component, eIF4G (Tarun and Sachs 1996; Tarun et al. 1997; Wells et al. 1998). In addition to stimulating mRNA translation in general, Pab1 inhibits the decapping of mRNAs in the cell (Coller and Parker 2004; Parker 2012). As expected, expression of Pab1 is tightly regulated. In this study, we demonstrate that the two RRM domains of Sbp1 bind specifically to the A-rich region in the 5′UTR of Pab1 (5′UTR_Pab1_) and the RGG domain of Sbp1 directly interacts with Pab1. These molecular interactions explain at least in part how Sbp1 inhibits the translation initiation of Pab1 mRNA.

## RESULTS

### Sbp1 binds to 5′UTR_Pab1_ in translation initiation

To see whether Sbp1 binds to 5′UTR_Pab1_ in the translation initiation pathway of the Pab1 mRNA, we developed a so-called “TRAP” assay. The assay uses molecules known to participate in initiation, including 5′UTRs, translational factors, and 40S ribosomal subunit, as a bait complex for an affinity pull-down procedure followed by mass spectrometry to identify the cellular proteins that interact with these intermediates in initiation. In our experimental design, proteins belonging to the class IV initiation factor family, which includes eukaryotic initiation factors eIF4E, eIF4G, eIF4A, and eIF4B, as well as eIF3 and eIF5, are not used in the bait. These proteins are known to participate in mRNA and 40S activation prior to the start-codon recognition (Jackson et al. 2010), thus their binding to bait complexes provides a valuable positive control for our pull-down results.

We cloned 5′UTR_Pab1_ and its sequence complement, 5′UTR_Pab1-complement_, as PCR-generated BamHI–HindIII fragments into the pTRAPv5 vector (Cytostore Inc.) downstream from the two streptavidin-binding S1 aptamers (Srisawat and Engelke 2001). Vectors were linearized with HindIII. Uncapped and aptamer-tagged RNA were transcribed from the DNA templates using T7 polymerase and purified by denaturing polyacrylamide gel electrophoresis or anion-exchange chromatography (Easton et al. 2010). The bait complex that we used in this assay corresponds to the 43S pre-initiation complex (43S PIC) that consists of a ternary complex (eIF2•GTP•Met-tRNA_i_^Met^) and a 40S ribosomal subunit (Jackson et al. 2010). Since binding of eIF1 and eIF1A to the 40S ribosome facilitates the recruitment of the ternary complex (Lorsch and Dever 2010), we also included eIF1 and 1A in the bait. Furthermore, we used the nonhydrolyzable GTP analog GDPCP to stabilize the ternary complex in the GTP state.

First, a stable formation of a 43S PIC on the 5′UTR_Pab1_ was confirmed by an electrophoresis mobility shift assay (EMSA) (Supplemental Fig. S1A). Next, the 5′UTR alone and 5′UTRs with 40S and initiation factors assembled were immobilized onto the streptavidin-coated magnetic beads and were incubated with yeast cell extracts. Following an extensive wash to remove most of the material bound nonspecifically, proteins that bind to the 5′UTR were eluted with D-biotin and analyzed by mass spectrometry. Using this assay, we confirmed that Sbp1 specifically binds to 5′UTR_Pab1_ (Supplemental Fig. S1B). At the same time, we also pulled down Pab1 protein from the cell extract using this assay (Supplemental Fig. S1B, lanes 3,5, and 7). This result is in good agreement with earlier findings that Pab1 binds to the polyadenosine sequences in its own 5′UTR (Sachs et al. 1987).

### Sbp1 binds to the 5′UTR_Pab1_ specifically at the poly(A) region

To test the possibility of a direct interaction between Sbp1 and the 5′UTR_Pab1_, purified Sbp1 (Supplemental Fig. S2A) and in vitro-transcribed 5′UTR_Pab1_ were combined and their interactions were examined by EMSA. As shown in Figure 1B, a complex is formed when Sbp1 and 5′UTR_Pab1_ are combined. Multiple bands are not seen at higher protein concentrations, suggesting that binding of Sbp1 and 5′UTR_Pab1_ is at 1:1 stoichiometry.

We further demonstrate that the interactions between 5′UTR_Pab1_ and Sbp1, which leads to the formation of the complex, are specific to each other. In a competition assay, the shifted band is efficiently outcompeted by an excess of unlabeled 5′UTR_Pab1_ (Fig. 1C, lanes 3 and 4) but is not affected by an excess of unlabeled 5′UTR_Pab1-complement_ with nucleotide sequence complementary to 5′UTR_Pab1_ (Fig. 1C, lanes 7 and 8). These results suggest that the observed band shifts are the result of formation of a specific, protein–RNA complex.

#### Domains of Sbp1 bind to the 5′UTR_Pab1_ cooperatively

To see if the domains of Sbp1 bind to the 5′UTR_Pab1_ cooperatively, we studied binding properties of individual domains of Sbp1 to 5′UTR_Pab1_ and compared the results with the wild-type Sbp1. Care was taken to ensure that individual domains—RRM1, RGG, and RRM2 domains of Sbp1 (Sbp1_RRM1_, Sbp1_RGG_, and Sbp1_RRM2_)—were folded properly (Supplemental Fig. S2B). As shown in Figure 1D, while individual domains of Sbp1 bind to the 5′UTR_Pab1_, they interact with the RNA with much weaker affinities compared to the affinity of the full-length Sbp1 with the 5′UTR_Pab1_. The measured apparent dissociation constant (*K*_d_) of full-length Sbp1 to the 5′UTR_Pab1_ is 26.5 ± 1.1 nM with a Hill coefficient of 2.1 ± 0.1 (Supplemental Fig. S3A). In contrast, under the same experimental conditions, the *K*_d_ values of its domain mutants, the Sbp1_RRM1_, Sbp1_RRM2_, and Sbp1_RGG_ to the 5′UTR_Pab1_ are about 1.1 μM, 0.9 μM, and 2.7 μM, respectively (data not shown). Thus, the binding of full-length Sbp1 to 5′UTR_Pab1_ is over 40 times stronger than that of its individual domains to the same RNA. These results demonstrate convincingly that the domains of Sbp1 bind to the 5′UTR_Pab1_ cooperatively.

#### Sbp1 binds to the internal poly(A) region in 5′UTR_Pab1_

**FIGURE 2.**
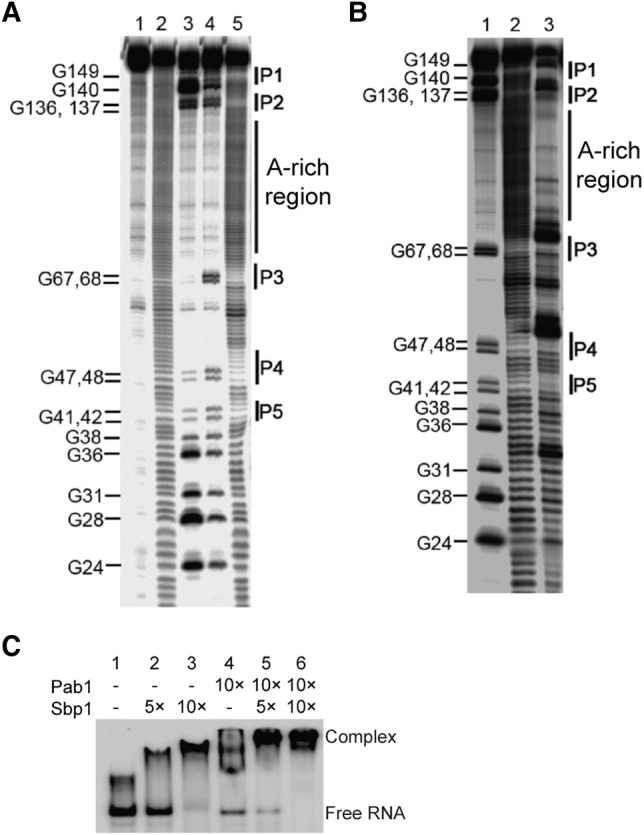
Sbp1 binds to the A-rich region in 5′UTR_Pab1_. (*A*) An in-line probing gel image of 5′UTR_Pab1_. The 5′UTR_Pab1_ in its undigested form (lane *1*) was subjected to partial alkaline digestion (lane *2*) and RNase T1 treatment in the presence of urea at 37°C (lane *3*) and at 55°C (lane *4*). Results on in-line probing of 5′UTR_Pab1_ (lane *5*) are shown. Double-stranded regions (P1, P2, P3, P4, and P5) and poly(A)-rich region in the RNA are shown. (*B*) An in-line probing gel image of 5′UTR_Pab1_ in the presence of Sbp1. 5′UTR_Pab1_ RNA partially digested by RNase T1 at 55°C in the presence of urea (lane *1*). The cleavage pattern of 5′UTR_Pab1_ after the in-line probing without Sbp1 (lane *2*) and with Sbp1 (lane *3*) showing protection of the A-rich region by Sbp1. (*C*) An EMSA showing the formation of 5′UTR_Pab1_•Sbp1•Pab1 complex. Addition of Sbp1 or Pab1 leads to formation of the RNP complexes of 5′UTR_Pab1_•Sbp1 (lanes *2* and *3*) or 5′UTR_Pab1_•Pab1 (lane *4*), respectively, and results in a slower electrophoretic mobility of the ^32^P-labeled 5′UTR_Pab1_. In the presence of Sbp1 and Pab1 simultaneously, a band that migrates even slower appears, suggesting the formation of 5′UTR_Pab1_•Sbp1•Pab1 complex (lanes *5* and *6*). Lane *1* shows 5′UTR_Pab1_ only.

To study how the 5′UTR_Pab1_ folds and which region in the RNA binds to Sbp1, we determined the secondary structures of 5′UTR_Pab1_ and 5′UTR_Pab1_–Sbp1 complex using in-line probing (Soukup and Breaker 1999). In-line probing is an RNA structure analysis technique that uses the relative flexibility of RNA backbone conformation to determine secondary structure characteristics of the RNA. As shown in Figure 2, in the absence of Sbp1, the A-rich region in the 5′UTR_Pab1_ adopts a single-stranded conformation (Fig. 2A). Addition of Sbp1 protects the degradation of the RNA in this region (Fig. 2B), suggesting that the protein binds to the A-rich sequence in the 5′UTR_Pab1_ (Supplemental Fig. S4).

As mentioned in the “Domains of Sbp1 bind to the 5′UTR_Pab1_ cooperatively” section, the *K*_d_ of Sbp1 to the 5′UTR_Pab1_ is 26.5 ± 1.1 nM. Under the same experimental conditions, including ionic strength of binding buffers, the *K*_d_ of Pab1 to the 5′UTR_Pab1_ is 31.2 ± 2.5 nM (Supplemental Fig. S3). These results show that Sbp1 and Pab1 bind to the A-rich region in the 5′UTR_Pab1_ with a similar affinity in vitro.

Since Pab1 binds to the poly(A) region in the 5′UTR_Pab1_ (Sachs et al. 1987) and both proteins are pulled down simultaneously on the 5′UTR_Pab1_ with the 43S PIC in our TRAP assay (Supplemental Fig. S1B), these results suggest that Sbp1 and Pab1 likely bind to the 5′UTR_Pab1_ at the same time. Indeed, a higher-order complex of 5′UTR_Pab1_–Sbp1–Pab1 is formed under our experimental conditions, as shown in a native EMSA (Fig. 2C).

### The RGG domain of Sbp1 directly interacts with Pab1 in an RNA-dependent manner

**FIGURE 3.**
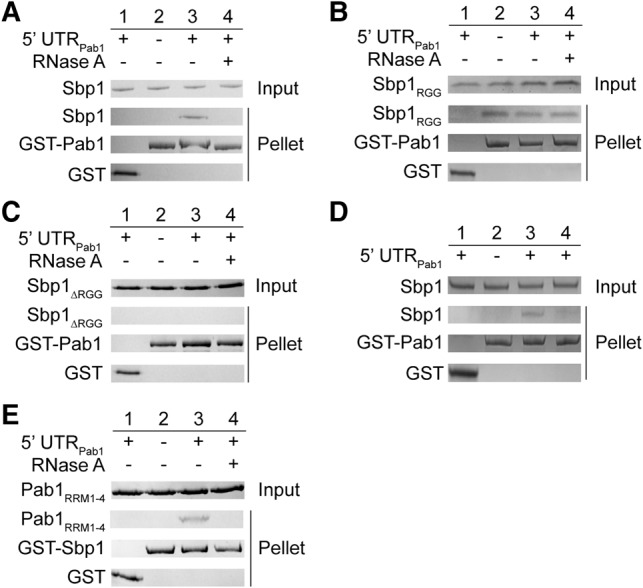
Sbp1 interacts with Pab1 in an RGG- and RNA-dependent manner. (*A*–*C*) GST-pulldown assays showing interactions of GST-tagged Pab1 with Sbp1 (*A*), Sbp1_RGG_ (*B*), and Sbp1_ΔRGG_ (*C*). Sbp1_RGG_ stands for RGG domain of Sbp1; Sbp1_ΔRGG_ stands for mutant with RGG domain replaced by glycine–serine repeats. Purified proteins were incubated in the absence of 5′UTR_Pab1_ (lane *2*), with 10× 5′UTR_Pab1_ (lane *3*) or with 10× 5′UTR_Pab1_ followed by RNase treatment (lane *4*). The proteins pulled down are shown by SDS–PAGE. Lane *1* shows the negative control containing GST only. (*D*) RNA-binding of Sbp1, not Pab1, is important for the Sbp1–Pab1 interaction. GST-pulldown assays showing Sbp1–Pab1 interaction under different conditions: Sbp1 incubated with Pab1 in the absence of 5′UTR_Pab1_ (lane *2*), GST-tagged Pab1 incubated with the Sbp1•5′UTR_Pab1_ complex (lane *3*), and Sbp1 incubated with the GST-Pab1•5′UTR_Pab1_ complex (lane *4*). In lane *4*, 5′UTR_Pab1_ was incubated with GST-Pab1 first, and unbound RNA in excess was removed prior to the addition of Sbp1. Lane *1* shows negative control with GST only. (*E*) Deletion of the C-terminal domain of Pab1 does not affect Sbp1–Pab1 interaction. GST-pulldown assay was performed for GST-Sbp1 and the C-terminal deleted variant Pab1 (Pab1_RRM1-4_). Sbp1 and Pab1_RRM1-4_ interact with each other in the presence of 5′UTR_Pab1_ (cf. lanes *2* and *3*), and RNase treatment disrupts the protein interaction (cf. lanes *3* and *4*). Proteins were incubated with GST as the negative control (lane *1*).

To test whether there is a direct interaction between Sbp1 and Pab1, we performed a glutathione *S*-transferase (GST) pull-down assay using purified GST-Pab1 and Sbp1 proteins. As shown in Figure 3A (lane 3), Sbp1 is pulled down by Pab1 only in the presence of 5′UTR_Pab1_ RNA. Addition of RNase A abolishes the protein–protein interaction (Fig. 3A, cf. lanes 3 and 4), suggesting that the interaction between Pab1 and Sbp1 is RNA-dependent.

In contrast, the RGG domain of Sbp1, Sbp1_RGG_, interacts with Pab1 directly in the absence of 5′UTR_Pab1_ (Fig. 3B, lane 2). Unsurprisingly, neither the presence of an equal stoichiometric amount of 5′UTR_Pab1_ nor an addition of RNase A in the presence of 5′UTR_Pab1_ alters the protein–protein interaction (Fig. 3B, cf. lanes 2, 3, and 4). A replacement of the RGG domain in Sbp1 with 17 glycine–serine repeats (GS repeats) abolishes the interaction between Sbp1 and Pab1 regardless of the presence of 5′UTR_Pab1_ (Fig. 3C, lanes 2, 3, and 4). Furthermore, Pab1 interacts with the Sbp1–5′UTR_Pab1_ complex (Fig. 3D, lane 3), whereas Sbp1 does not interact with the Pab1–5′UTR_Pab1_ complex (Fig. 3D, lane 4). These observations demonstrate that the RNA binding of Sbp1, not Pab1, is important for interactions between Pab1 and Sbp1. As such, our results indicate that exposing the RGG domain of Sbp1 by the RNA binding is required for Sbp1 to bind Pab1.

Pab1 in yeast is a 71 kDa RNA-binding protein with four RNA recognition motifs (RRM1–4) and a C-terminal protein-binding domain, also known as PABC (Wigington et al. 2014). To determine which regions in the Pab1 interact with Sbp1, we generated domain mutants of Pab1 and tested their bindings with Sbp1. Our results show that the protein construct containing the RRM1–4 domains of Pab1, Pab1_RRM1–4_, binds to Sbp1 (Fig. 3E). In contrast, no molecular interaction was detected when PABC, or individual RRM domain mutants including Pab1_RRM1_, Pab1_RRM2_, Pab1_RRM3_, and Pab1_RRM4_ as well as other tandem RRM domains, were tested (Supplemental Fig. S5A–H). These observations, suggest that, together, RRM1–4 domains in Pab1 are responsible for the interaction with Sbp1. Consistent with our previous observations, Sbp1 and the RRM1–4 of Pab1 only interact with each other in the presence of 5′UTR_Pab1_ (Fig. 3E, cf. lanes 2 and 3), and addition of RNase A abolishes the protein–protein interaction (Fig. 3E, cf. lanes 3 and 4). Taken together, these results confirm that molecular interactions between Pab1_RRM1-4_ and Sbp1 are RNA-dependent: Binding of the RNA is important to expose the RGG repeats of Sbp1, which is required for the interaction with Pab1_RRM1–4_.

### Formation of a stable 5′UTR_Pab1_–Sbp1–Pab1–eIF4G1 RBP in vitro

**FIGURE 4.**
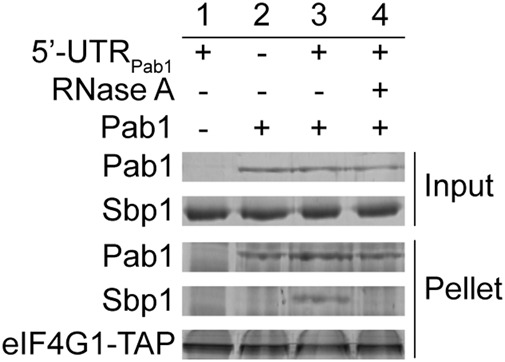
Sbp1, Pab1, and eIF4G1 interact with each other and formation of the ternary complex is RNA-dependent. IgG-pulldown assay showing that Sbp1, Pab1, and eIF4G1 interact in the presence of 5′UTR_Pab1_ (cf. lanes *2* and *3*), and the molecular interaction is disrupted by RNase treatment (cf. lanes *3* and *4*). Lane *1* shows that Sbp1 and eIF4G1 do not interact with each other when Pab1 is absent.

Thus far, we demonstrated that Sbp1 interacts with Pab1 via its RGG domain in an RNA-dependent manner. It is known that the RRM2 domain of Pab1 binds to eIF4G1 directly (Kessler and Sachs 1998; Safaee et al. 2012). Furthermore, earlier mass spectrometry results show that Sbp1, Pab1, and eIF4G interact with each other (Gavin et al. 2002, 2006). To test formation of a stable 5′UTR_Pab1_–Sbp1–Pab1–eIF4G1 RBP in vitro, we did an IgG-pulldown assay using purified eIF4G1, Pab1, and Sbp1. In this experiment, in order to ensure the correct folding and functionality of the protein, eIF4G1 was chromosomally expressed and affinity-purified in yeast. Furthermore, endogenous RNAs that bind to eIF4G1 and Pab1 were removed (see Materials and Methods) to minimize interference of the bound RNAs to protein–protein interactions. As shown in Figure 4 (cf. lanes 2 and 3), the three proteins bind simultaneously only in the presence of 5′UTR_Pab1_. As expected, formation of the ternary complex (Sbp1–Pab1–eIF4G1) on the 5′UTR_Pab1_ was disrupted by the addition of RNase A (Fig. 4, cf. lanes 3 and 4), suggesting the importance of the RNA in the ternary complex formation.

### Methylation of RGG domain compromises the Sbp1-protein binding but leaves the Sbp1–RNA binding unchanged

Methylation at the guanidino group in the arginine side chain, a eukaryotic-specific modification, has been suggested as one of the most extensive protein methylations in mammalian cells (Najbauer et al. 1993; McBride and Silver 2001; Pahlich et al. 2006; Bedford and Clarke 2009; Blanc and Richard 2017). This modification plays important physiological roles from DNA repair, transcriptional regulation, mRNA splicing, to protein translocation and signal transduction. Sbp1 is a known target for the arginine methyltransferase (Hmt1) (Frankel and Clarke 1999), but the physiological function of this modification remains unknown.

**FIGURE 5.**
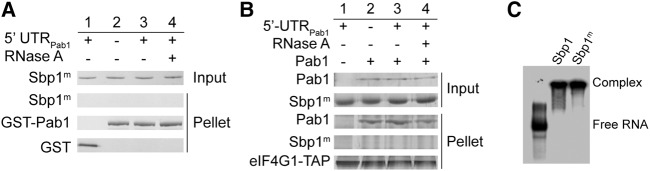
Methylation of RGG domain disrupts protein–protein but not protein–RNA interactions. (*A*) GST-pulldown assay showing RGG methylation in Sbp1 (Sbp1^m^) disrupts the Sbp1–Pab1 interaction. Sbp1^m^ was incubated with GST-Pab1 without 5′UTR_Pab1_ (lane *2*), in the presence of 10× 5′UTR_Pab1_ (lane *3*) or RNase A (lane *4*). The protein was incubated with GST as a negative control (lane *1*). (*B*) IgG-pulldown assay showing Sbp1^m^ compromises ternary complex formation. Sbp1^m^ and Pab1 were incubated with eIF4G1-TAP without 5′UTR_Pab1_ (lane *2*), in the presence of 10× 5′UTR_Pab1_ (lane *3*), or an incubation followed by RNase A treatment (lane *4*). Sbp1^m^ and eIF4G1 show no interaction in the presence of 5′UTR_Pab1_ (lane *1*). (*C*) EMSA showing that methylation of RGG repeats in Sbp1 does not affect RNA binding. ^32^P-labeled 5′UTR_Pab1_ was incubated with 10× Sbp1 (lane *2*) or 10× methylated Sbp1 (lane *3*). Lane *1* is 5′UTR_Pab1_ alone.

To study roles of the arginine methylation in mediating protein–protein as well as protein–RNA interactions, we first obtained Sbp1 with the methylated RGG domain by coexpressing Sbp1 with its cognate methyltransferase Hmt1 (Supplemental Fig. S2A; Hsieh et al. 2007). We then confirmed methylation of arginines in the RGG domain by mass spectrometry (Supplemental Fig. S6). Next, using the same GST-pulldown of purified proteins and EMSA as described in the previous sections, we demonstrated that RGG methylation (RGG^m^) compromises the binding of Sbp1 to Pab1 (Fig. 5A, cf. lanes 3 and 4) and to the formation of the ternary complex (Sbp1–Pab1–eIF4G1) on the 5′UTR_Pab1_ (Fig. 5B, cf. lanes 3 and 4). In contrast, methylation of the RGG domain has no influence on the binding of Sbp1 to 5′UTR_Pab1_ (Fig. 5C, cf. lanes 2 and 3).

### Sbp1 inhibits both cap-dependent and cap-independent translation initiation of Pab1 mRNA in an RGG- and poly(A)-dependent manner

To test the functional importance of these molecular interactions in transcript-specific translation initiation, we carried out in vitro translation assays using luciferase reporter genes (Hofbauer et al. 1982; Hussain and Leibowitz 1986; Wang et al. 2013). We generated a monocistronic reporter construct by cloning the 5′UTR of Pab1 mRNA in-frame with the firefly luciferase open reading frame (ORF) under control of the T7 promoter. Cap-dependent and cap-independent translation, the two pathways for the translation initiation of eukaryotic mRNAs, can be distinguished by adding or omitting the 5′ m^7^G cap to the mRNAs under study. Furthermore, to inhibit ribosome scanning, a stable hairpin upstream of the uncapped 5′UTR_Pab1_ was used to study the cap-independent translation initiation (Wang et al. 2013) in our investigation. The same strategy was used to generate the monocistronic reporter constructs for the 5′UTR of eIF4E mRNA as a control. Additionally, RNAs with nucleotide sequence complementary to 5′UTR_Pab1_ and 5′UTR_4E_, 5′UTR_Pab1-complement_ and 5′UTR_4E-complement_, were used as controls to rule out nonspecific interactions. The same amount of RNA was added in each translation assay and all measured activities were normalized to the total protein level in cell extracts. Finally, translation of cricket paralysis virus (CrPV) depends on correct folding of the 5′UTR of the CrPV RNA (5′UTR_CrPV_, also known as the internal ribosome entry site of CrPV) alone and does not require any additional proteins (Thompson et al. 2001). As such, 5′UTR_CrPV_ was included as an experimental control.

**FIGURE 6.**
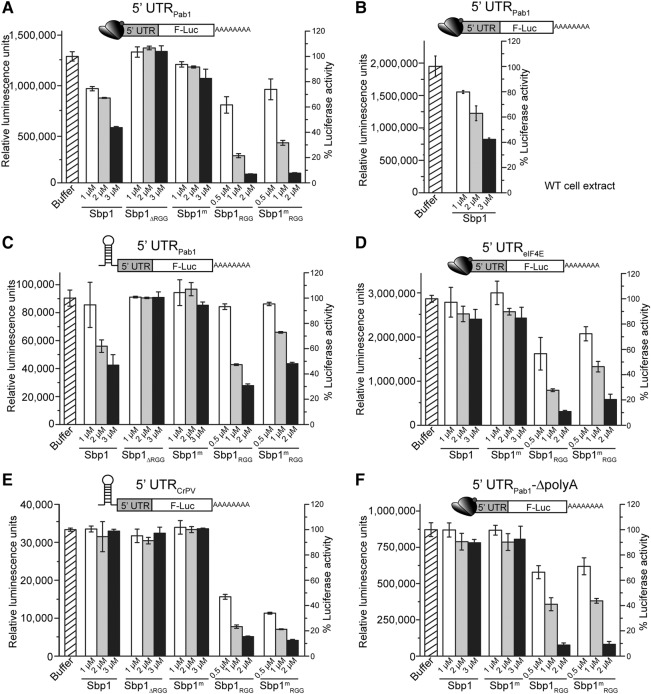
Sbp1 inhibits both cap-dependent and cap-independent translation initiation of Pab1 mRNA in an RGG- and RNA-dependent manner. Reporter constructs are shown schematically in the *top* panel of the figure. In vitro cap-dependent and cap-independent translation activities of 5′UTR_Pab1_ and 5′UTR_Pab1-Δpoly(A)_ in the presence of different concentrations of Sbp1 and its variants are shown. (*A*) Changes of cap-dependent translation initiation activities of 5′UTR_Pab1_ by Sbp1 and its protein variants. In vitro translation assays were performed by incubating the capped 5′UTR_Pab1_ followed by firefly luciferase gene and poly(A) tail [5′UTR_Pab1_-FLuc-poly(A)] in ΔSbp1 cell extract with varying amounts of Sbp1, Sbp1_ΔRGG_, Sbp1^m^, Sbp1_RGG_, and Sbp1^m^_RGG_ added. Sbp1_ΔRGG_: a substitution of RGG domain with glycine–serine repeats; Sbp1^m^: methylated Sbp1; Sbp1_RGG_: RGG domain of Sbp1; Sbp1^m^_RGG_: methylated RGG domain. (*B*) Changes of cap-dependent translation initiation activities of 5′UTR_Pab1_ at varying concentrations of Sbp1 added to WT cell extracts. (*C*) Changes of cap-independent translation initiation activities of 5′UTR_Pab1_ by Sbp1 and its protein variants. Experiments were done as in *A* using a different reporter construct, the uncapped 5′UTR_Pab1_-FLuc-poly(A), which has a hairpin insertion before the 5′UTR_Pab1_ to inhibit ribosome scanning. (*D*) Changes of cap-dependent translation initiation activities of 5′UTR_4E_ by Sbp1 and its protein variants. Experiments were done as in *A*, except the capped 5′UTR_eIF4E_-FLuc-poly(A) was used. (*E*) Changes of cap-independent translation initiation activities of 5′UTR_CrPV_ by Sbp1 and its protein variants. Experiments were done as in *A*, except the uncapped Hairpin-5′UTR_CrPV_-FLuc-poly(A) was used. (*F*) Changes of cap-dependent translation initiation activities of 5′UTR_Pab1-Δpoly(A)_ by Sbp1 and its protein variants. 5′UTR_Pab1-Δpoly(A)_: 5′UTR_Pab1_ RNA with the internal A-rich region deleted. The integrity of the RNAs was confirmed by either northern blot or ethidium bromide-stained gels under denaturing conditions. Luciferase activities are normalized to the amount of capped or uncapped RNAs and the total protein levels in the study. Reported translation activities are the average of results obtained in three independent experiments. Controls including translation activities of the blank, 5′UTR of eIF4E (5′UTR_eIF4E_) and internal ribosome entry site of cricket paralysis virus (5′UTR_CrPV_) under the same experimental conditions are shown in *D* and *E*.

A capped 5′UTR_Pab1_ or an uncapped 5′UTR_Pab1_ with a stable upstream hairpin was used for measuring the cap-dependent or cap-independent initiation activity, respectively. These RNA constructs were incubated with the yeast extracts from either WT or ΔSbp1 cells with an increasing amount of Sbp1 protein added. As shown in Figure 6A and C (the second column), a decreased translation activity in the presence of an increasing amount of Sbp1 indicated an inhibitory function of this protein in both cap-dependent and cap-independent initiation of the Pab1 mRNA. In contrast, translation of the 5′UTR_4E_ as well as 5′UTR_CrPV_ was almost unchanged as the concentration of Sbp1 increases (Fig. 6D,E).

Since Sbp1 coimmunoprecipitates with small nucleolar RNA 10 and 11 (Jong and Campbell 1986; Jong et al. 1987; Clark et al. 1990), to rule out possibilities of a change in mRNA translation due to changes of ribosome biogenesis by Sbp1 in the cell, we added purified Sbp1 into the WT cell extract and observed a similar inhibitory function of the translation of Pab1 mRNA by Sbp1 (Fig. 6B).

Importantly, a substitution of the RGG repeats of Sbp1 with GS repeats (column 2 in Fig. 6A,C), or methylation of the RGG domain (column 3 in Fig. 6A,C), or deletion of the internal poly(A) sequence (Fig. 6F, columns 2 and 3), diminishes the inhibitory effect of Sbp1 on the Pab1 mRNA. Taken together, our results show that both the RGG domain and poly(A)-sequence of 5′UTR_Pab1_ are important for Sbp1's function in translation of the Pab1 mRNA.

## DISCUSSION

Sbp1 is a single-stranded RNA-binding protein that contains two RNA recognition motifs and an arginine–glycine–glycine rich domain. In this study, we investigated molecular interactions important for the transcript-specific translational regulation of Sbp1 on an essential mRNA that encodes the poly(A)-binding protein Pab1 in vitro. Our results demonstrate that the RNA-binding property and RGG domain of Sbp1 are important for its function.

### Sbp1 binds to the poly(A) region in 5′UTR_Pab1_ specifically

We show that the two RRMs of Sbp1 bind to the poly(A) region of 5′UTR_Pab1_ specifically, and this protein–RNA interaction is important for the inhibition of translation initiation of Pab1 mRNA. Four observations support this conclusion. First, direct binding of Sbp1 to 5′UTR_Pab1_ was shown by a TRAP assay where Sbp1 was pulled down from cell lysates using 5′UTR_Pab1_ alone and 5′UTR_Pab1_ assembled with a preformed 43S PIC (Supplemental Fig. S1B). Consistent with this result, a recent genome-wide, cross-linking study shows that Sbp1 preferentially binds to the 5′UTRs of mRNAs (Mitchell et al. 2013). Second, 5′UTR_Pab1_, but not its sequence complement, 5′UTR_Pab1-complement_, competes for the binding of Sbp1 (Fig. 1). Third, binding analyses based on EMSAs reveal that the individual domains of Sbp1, while folded properly, have much weaker affinities for the 5′UTR_Pab1_ than the full-length protein and, therefore, suggests that the domains in the protein act cooperatively for RNA binding.

Fourth, results from our in-line probing experiments on 5′UTR_Pab1_ alone (Fig. 2A) and the 5′UTR_Pab1_–Sbp1 complex (Fig. 2B) show that the A-rich region in 5′UTR_Pab1_ is protected upon the binding of Sbp1, indicating that this region in 5′UTR_Pab1_ is likely to be the major binding site of Sbp1 (Supplemental Fig. 4). The measured *K*_d_ of Sbp1 and 5′UTR_Pab1_ is 26.5 ± 1.1 nM. Templated poly(A)-rich tracts have been found in UTRs of many cellular mRNAs in eukaryotes including yeast and humans (Gilbert et al. 2007; Wigington et al. 2014). It is conceivable that Sbp1 likely binds to the A-rich region in these mRNAs in a similar way.

It has been shown that the first two RRM domains of the Pab1 bind to the polyadenosine sequence in the 5′UTR_Pab1_ with nanomolar affinity (Sachs et al. 1987; Kessler and Sachs 1998), which contributes to the mechanism of an autoregulated translation of Pab1 mRNA in vitro and in vivo (de Melo Neto et al. 1995; Bag and Wu 1996; Hornstein et al. 1999). The apparent *K*_d_ of the 5′UTR_Pab1_ and Pab1 is 31.2 ± 2.5 nM (Supplemental Fig. S3), which is slightly higher compared to that obtained on a 5′UTR_Pab1_–Sbp1 complex (26.5 ± 1.1 nM) under the same experimental conditions.

### The RNA interaction and RGG domain are important for an inhibited translation initiation by Sbp1 on Pab1 mRNA

As shown in Figure 6A, C, and F, an increasing amount of Sbp1 leads to a 20%–60% decrease in the cap-dependent translation initiation activity of 5′UTR_Pab1_. Similar changes in the cap-independent activity of 5′UTR_Pab1_ were also observed. In contrast, the cap-dependent translation initiation activities of 5′UTR_Pab1-Δpoly(A)_ remain nearly unchanged when the concentration of Sbp1 increases.

In addition to RNA binding, the RGG domain of Sbp1 is important for the function of Sbp1. Substitution of the RGG repeats with GS repeats, or methylation of arginines in the RGG repeats, abolishes the inhibitory function of Sbp1 on the Pab1 mRNA in both cap-dependent and cap-independent initiation pathways (Fig. 6A,C).

### RGG domain of Sbp1 binds to Pab1, and formation of the binary and ternary protein complex on the 5′UTR_Pab1_ is RNA-dependent

To rule out the possibility that RNA or a tertiary factor present in cell extracts mediates the protein–protein interaction, we carried out the pulldown assay using purified proteins such as Sbp1 and Pab1 (Supplemental Fig. S2A). Heparin column and stringent multiple washing procedures were used to eliminate residual RNAs associated with these RNA-binding proteins (see Materials and Methods). Under our experimental conditions, Sbp1 directly binds to initiation factors Pab1 via its central RGG domain. Importantly, the RGG domain of Sbp1, not the RRM domains, is required for the protein–protein interactions, as demonstrated by results obtained from the pulldown assay using domain mutants of Sbp1 (Fig. 3B,C). We further showed that four RRM domains in Pab1 (RRM1-4) are required for the binding with Sbp1. Consistent with our results, Sbp1 was identified to associate with Pab1 in a recent study (Richardson et al. 2012).

Pab1 and eIF4G were known to interact with each other (Tarun and Sachs 1996; Tarun et al. 1997; Kessler and Sachs 1998). Furthermore, Sbp1 was shown to interact with eIF4G1, eIF4G2, and Pab1 in the cell in a proteomic investigation using open-reading frame tagging, affinity purification, and mass spectrometry (Gavin et al. 2002, 2006). It is conceivable that higher-order complexes can be formed on RNA by these proteins. Indeed, interactions between Sbp1, Pab1, and eIF4G1 on 5′UTR_Pab1_ were observed (Fig. 4).

Importantly, addition of RNase disrupts the Sbp1–Pab1 as well as Sbp1–Pab1–eIF4G1 interactions on 5′UTR_Pab1_ (Figs. 3A, 4), but leaves Sbp1_RGG_–Pab1 and Pab1–eIF4G1 interactions unchanged (Figs. 3B, 4). These results confirm that the exposure and conformation of the RGG domain in Sbp1 by RNA binding is important for the protein–protein interaction, which explains why the interaction between Sbp1–Pab1 is RNA-dependent. Furthermore, we noticed that the RGG repeats in Sbp1 are located in between the two RRM domains, much unlike many other RGG-motif proteins which contain the RGG repeats in the terminal regions (data not shown). This domain organization of Sbp1 would likely remodel the RNA structure that it binds by positioning of the central RGG repeats for protein interactions in an RNA-dependent manner.

It is noteworthy that the interaction between Sbp1 and eIF4G1 is mediated by Pab1 on 5′UTR_Pab1_. Using purified RNA-free proteins, we observed no direct interactions between eIF4G1 and Sbp1, and between eIF4G1 and the RGG domain of Sbp1 (Sbp1_RGG_) (Supplemental Fig. S7). Previously, Sbp1 was shown to interact with translation initiation factor eIF4G1 via its RGG domain by a GST-pulldown assay using overexpressed eIF4G1 in *E. coli* cell extracts (Rajyaguru et al. 2012). We reasoned that the observed interaction between Sbp1 and eIF4G1 under the described experimental condition is likely mediated by endogenous RNAs or a ternary protein.

### Arginine methylation of Sbp1 compromises its interactions with Pab1

Post-translational modifications of the RGG domain of Sbp1 were known to modulate its molecular interactions in the cell (Hsieh et al. 2007). In this study, we demonstrated that arginine methylation in the RGG repeats of Sbp1 compromised the binding of Sbp1 to Pab1. In contrast, the same post-translational modification has little effect on the binding of Sbp1 to the RNA (5′UTR_Pab1_) in vitro. Results from our in vitro translation assay show that the methylation of the RGG domain abolishes the translation repression activity of Sbp1 on 5′UTR_Pab1_.

Arginine methylation has been known to be important in changing protein–protein interactions as well as in controlling subcellular localization of several proteins (Shen et al. 1998; Sinha et al. 2010). Furthermore, the expression of Hmt1 is cell cycle-dependent and the protein is active under different growth conditions in yeast (Messier et al. 2013), suggesting Sbp1 may play a role in the 5′UTR activation during cell cycle progression*.* Further investigations are needed to understand how the interactions between Sbp1 and Pab1, as well as the formation of a higher-order complex with eIF4G1 involved, are regulated by arginine methylation and the functional consequences of this regulation in translation of targeted mRNAs in the cell.

### A mechanistic model for translation regulation by Sbp1

**FIGURE 7.**
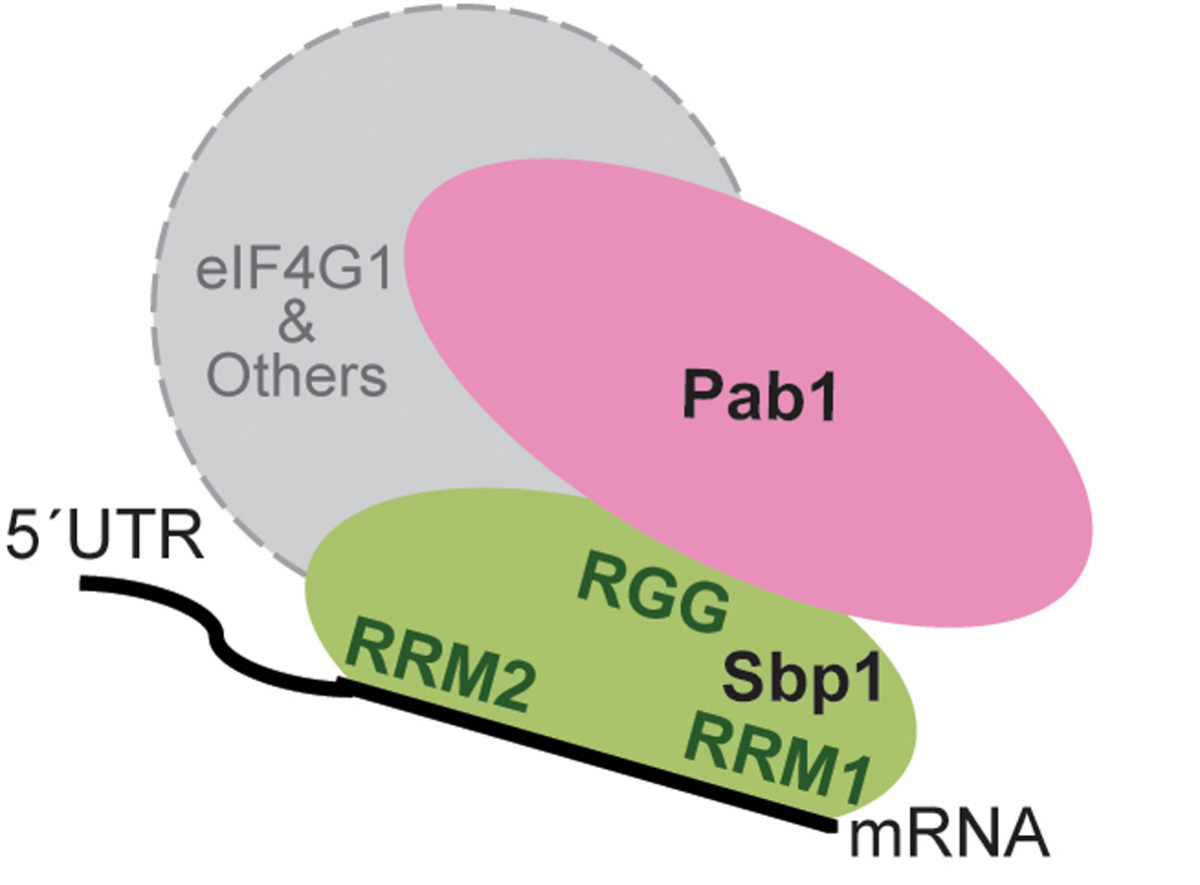
A proposed mechanism of Sbp1-specific translation regulation. The two RRM domains of Sbp1 bind to 5′UTRs cooperatively, and the center RGG domain recruits proteins such as initiation factor Pab1. These interactions remodel the 5′UTR assembly, leading to changes in the recruitment of the 43S PIC to the target mRNAs and the downstream translational events.

Based on the results, our mechanistic hypothesis for the Sbp1-specific translation regulation is the following (Fig. 7): The two RRM domains of Sbp1 bind to 5′UTRs cooperatively, and the center RGG domain recruits other proteins. These interactions lead to formation of an RNP assembly on the UTR, which modulates the translation of targeted mRNAs. In the case of 5′UTR_Pab1_, the RGG domain interacts with other proteins including Pab1, and the resulting RNP complex inhibits the translation initiation of Pab1 mRNA. Because Pab1 is a general translation factor, the observed down-regulation of this protein by Sbp1 may explain at least in part why Sbp1 inhibits the global translation of mRNAs in the cell.

**FIGURE 8.**
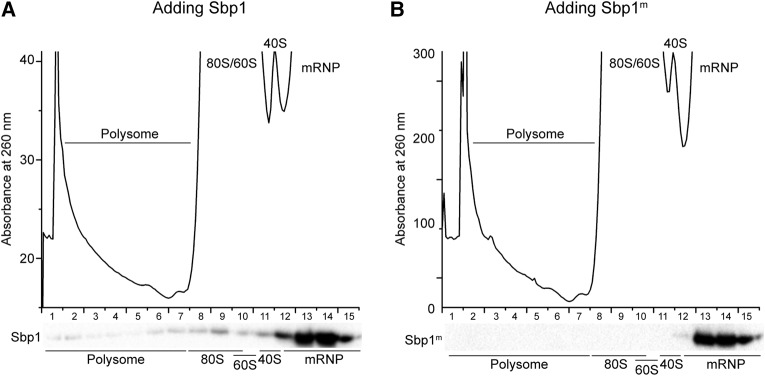
Methylation of the RGG repeats disrupts ribosome association of Sbp1. (*A*) Distribution of His-tagged Sbp1 proteins in the polysome profile of 5′UTR_Pab1_-FLuc-poly(A). (*B*) Distribution of His-tagged methylated Sbp1^m^ proteins in the polysome profile of 5′UTR_Pab1_-FLuc-poly(A). Capped 5′UTR_Pab1_ with luciferase reporter, 5′UTR_Pab1_-FLuc-poly(A), was incubated with ΔSbp1 cell extract in the presence of either 3 µM Sbp1 (A) or Sbp1^m^ (*B*). 10%–15% sucrose gradient was used for polysome profiling, and distribution of the protein was monitored by western blot. Peaks of polysome, 80S, 60S, 40S, and mRNA are indicated.

Consistent with this hypothesis on the importance of RNA binding, the distribution patterns of Sbp1 and Sbp1^m^ (arginine methylated Sbp1) in polysome profiles of 5′UTR_Pab1_ differ substantially, as shown in Figure 8. In this experiment, in vitro translations of 5′UTR_Pab1_ in-frame with the firefly luciferase reporter were carried out in the ΔSbp1 cell extracts in the presence of Sbp1 or methylated Sbp1^m^. Polysome profiles on the transcript were studied and presence of the protein in different fractions was detected by western blot (see Materials and Methods). As shown in Figure 8, Sbp1 mainly cosediments with RNP and 40S fractions. A small portion of the protein also cosediments with the 80S and polysome fractions. These results are consistent with the observation that tagged Sbp1 was pulled down with 80S and polysomes (Zhang et al. 2014; Wang et al. 2016).

In contrast, Sbp1^m^ only cosediments with the RNP fraction and does not show interactions with the ribosome. This result suggests that the methylation state of the RGG repeats in Sbp1 abolishes its interaction with the ribosome. In addition, because of changes in the protein–protein interaction by methylation, the nature of RNPs formed with Sbp1 and Sbp1^m^ on 5′UTR_Pab1_ is likely to be different.

Our study demonstrates that 5′UTR remodeling by RBP is important for the transcript-specific translation regulation. The RNA binding is critical for exposing Sbp1's central RGG repeats, which in turn is important for the molecular interactions between Sbp1, Pab1, and eIF4G1. In contrast, deletion of the RRM domain, whereby leaving the RGG domain alone, regardless of its methylation state, inhibits the translation of all the examined RNAs including Pab1, eIF4E, and CrPV in both cap-dependent and cap-independent pathways (Fig. 6, columns 5 and 6).

While our data strongly suggest a synergistic model described, we cannot, however, rule out a competitive binding of Sbp1 and Pab1 on the same RNA. Since both proteins can bind to the polyadenosine or A-rich sequences, they could potentially compete for the same target sequences. In this scenario, RNA remodeling is also likely to be accompanied when one protein replaces the other. Pab1 contains four tandem RRMs in which the first two RRM domains mediate the polyadenosine RNA binding (Sachs et al. 1987; Kühn and Pieler 1996; Deo et al. 1999; Safaee et al. 2012). Furthermore, a stretch of the RNA containing 12 consecutive adenosines or longer is required for a high affinity binding to Pab1 (Sachs et al. 1987). On the other hand, in the case of Sbp1, we showed that the protein mainly binds to the A-rich sequence in the RNA cooperatively using multiple domains, especially the two RRM domains flanking the central RGG repeats. Thus, the distinctive RRM domain architecture in Pab1 and Sbp1 will most likely lead to different conformations when these two proteins interact with the same RNA and, as a result, remodel the RNA structure differently. A related question is whether Sbp1 prefers binding to specific transcripts containing poly(A) or perhaps even the same transcript in different stages of mRNA biogenesis, considering that Sbp1 also binds to mRNA sequences other than poly(A) (Mitchell et al. 2013; Wang et al. 2016). Clearly, further studies involving genomic and structural investigations are required to answer these questions.

Recently, decapping activator Dhh1 has been shown to promote decapping by slowing ribosome movement on mRNA (Sweet et al. 2012). Since Sbp1 was suggested to stimulate decapping activity of mRNAs with Dhh1 (Segal et al. 2006) and we also observed that Sbp1 interacts with polysomes (Fig. 8), a fascinating question to ask next is whether, similar to Dhh1, Sbp1 also targets an elongating ribosome to control the translation of mRNA. Further investigations will be needed to provide a complete picture of Sbp1-specific translational regulation.

## MATERIALS AND METHODS

### Yeast strains and plasmid constructions

The YS602 strain from *Saccharomyces cerevisiae* (a generous gift from Professor Maurille J. Fournier from UMass Amherst) was used to generate Sbp1-depleted strain (ΔSbp1::kan) by homologus recombination using standard methods, as previously described (Baudin et al. 1993; Wach et al. 1994). Monocistronic luciferase reporters were generated by inserting the firefly luciferase gene between BamHI and KpnI restriction sites of the pUC18 vector (Clontech). A poly(A)_62_ was introduced between KpnI and EcoRI restriction sites downstream from the luciferase gene. 5′UTRs of Pab1 and eIF4E, including a 36-nt-long endogenous sequence in the open reading frame to maintain the AUG context, were cloned into XbaI and BamHI restriction sites of pUC18, in-frame and upstream of the luciferase gene. RNAs with sequence complementary to the 5′UTR_Pab1_ and 5′UTR_eIF4E_, termed 5′UTR_Pab1-complement_ and 5′UTR_eIF4E-complement_, were used as an RNA length and sequence control. The 5′UTR_Pab1-complement_ and 5′UTR_eIF4E-complement_ were generated in the same way as the 5′UTR_Pab1_. The 5′UTR_CrPV_ sequence was amplified from a pEJ1014 vector (a generous gift from Professor Eric Jan from the University of British Columbia) (Ren et al. 2012) and also cloned in the same way as the 5′UTR_Pab1_.

A T7 promoter sequence or the same sequence followed by a stable hairpin was cloned into the HindIII and XbaI restriction sites upstream of the 5′UTRs for the cap-dependent or cap-independent translation assays, respectively. The hairpin sequence (5′-CTGCAGCCACCACGGCCCAAGCTTGGGGG CCGTGGTGGCTGCAGGAGAGAGATTCC-3′) was used to inhibit ribosome scanning (Wang et al. 2013).

Genes encoding translation initiation factor eIF1, eIF1A, Pab1, Sbp1 and individual domains of Sbp1 and Pab1 were amplified from the genomic DNA of *S. cerevisiae* and cloned into the pETNKI-GST-LIC-Amp or pETNKI-His6-LIC-Amp vectors (Luna-Vargas et al. 2011). The type I arginine methyltransferase (*HMT1*) gene from *S. cerevisiae* and the eIF2 β and γ subunits were cloned into the pETNKI-His6-LIC-Kan vector. eIF2α was cloned into the pET22b vector. All the pETNKI vectors are generous gifts from Dr. Patrick Celie at the Netherlands Cancer Institute Protein Facility.

5′UTR_Pab1_ and 5′UTR_Pab1-complement_ were cloned into BamHI and HindIII restriction sites downstream from two streptavidin-binding S1 aptamers in the pTRAPv5 vector (Cytostore) for the TRAP assay.

### Protein expression and purification

His-tagged or GST-tagged eIF1, eIF1A, Sbp1, and Pab1 and their mutants containing individual domains were overexpressed in *E. coli* BL21 (DE3) with 0.1 mM isopropyl β-D-1-thiogalactopyranoside (IPTG) at 18°C. The proteins were purified by affinity chromatography using either Ni-NTA or glutathione agarose, which is followed by ion-exchange chromatography and HiTrap Heparin HP columns (GE Healthcare). Circular dichroism (CD) was performed on the wild-type Sbp1 and its individual RRM domains to confirm proper folding of the RRM domain mutants. eIF2 was expressed in yeast and purified as previously described (Acker et al. 2007). A TAP tag with a calmodulin binding peptide and two IgG binding domains of protein A (Dharmacon) was inserted at the end of the eIF4G1 gene in its chromosome locus. The C-terminal TAP-tagged eIF4G1 was expressed in yeast cells and purified with affinity chromatography using buffer containing 20 mM Hepes-KOH, pH 7.9, 150 mM KCl, 1 mM Mg(OAc)_2_, 0.1% NP-40, 1 mM DTT followed by a HiTrap Heparin HP column (GE Healthcare) using buffer containing 20 mM Tris–HCl, pH 7.5, 150 mM KCl, 0.1% NP-40. RNase A and high salt wash with 1 M KCl were used to remove endogenous RNAs bound to eIF4G1.

### In vitro transcription

RNAs were synthesized by run-off transcription from linearized DNA plasmid templates and purified by either gel electrophoresis under denaturing conditions or by anion-exchange chromatography (Easton et al. 2010). The purity and integrity of the RNA was monitored by an acrylamide gel under denaturing conditions or by northern blot.

### TRAP assay

All steps were performed in a buffer of 50 mM Hepes pH 7.3, 50 mM KCl, 10 mM Mg(C_2_H_3_O_2_)_2_, 2 mM DTT, 1 mM phenylmethylsulfonyl fluoride (PMSF), and 1 mM benzamide. Of note, 40 U RNase inhibitor (NEB) were added per 1 mg/mL of total protein concentration. Cell extracts from *S. cerevisiae* were precleared with 60 µL of paramagnetic streptavidin-conjugated magnetic beads (Dynabeads M-280; Invitrogen) for 1 h at 4°C to decrease the amount of protein bound nonspecifically to the beads. S1-tagged 5′UTR_Pab1_ RNAs alone and the 5′UTR_Pab1_-43S PIC were incubated with the beads for 2 h at 4°C in the buffer consisting of 50 mM Hepes, pH 7.3, 100 mM KCl, 2 mM MgCl_2_, and 2 mM DTT. The formation of the 43S PIC was carried out as described previously (Acker et al. 2007). To facilitate ribosome-loading, 5′UTR_Pab1-construct1_ was used in which the RNA sequence downstream from the start codon AUG was replaced by AAAAAAAAA.

Subsequently, RNA-bound streptavidin beads were incubated with cell extracts for 2 h at 4°C. Proteins bound to the 5′UTR assembly were eluted by D-biotin (Invitrogen), examined by sodium dodecyl sulfate polyacrylamide gel electrophoresis (SDS–PAGE) and identified and quantified by liquid chromatography–tandem mass spectrometry (LC–MS/MS).

### In vitro translation assays

Cells were harvested at OD_600_ = 0.85–0.9 and washed five times with translation buffer comprised of 22 mM Hepes-KOH, pH 7.4, 120 mM potassium acetate, 1.5 mM magnesium acetate, 0.75 mM ATP, 0.1 mM GTP, 25 mM creatine phosphate (Invitrogen), 0.04 mM of each of the twenty amino acids, 1.7 mM 1,4-dithiothreitol (DTT), 5 µg creatine kinase (Invitrogen), and 10 U RNasin Plus (Promega). Cell extracts were treated with micrococcal nuclease and prepared as previously described (Iizuka et al. 1994). An m^7^G cap was added at the 5′ end of the RNA using the Vaccinia capping enzyme (NEB). In vitro translation reactions were carried out at 25°C for 40 min in translation buffer. m^7^G-capped or uncapped RNAs were added to the extracts in the presence of Sbp1 and its domain variants at varying concentrations, and luciferase activities were measured using the ONE-Glo Luciferase Assay kit (Promega). The measured reporter activity was normalized to the concentration of total proteins in the extract obtained by Bradford assay. All reported activities are an average of at least three independent luciferase assays.

### Electrophoretic mobility shift assay

^32^P-labeled 5′UTR_Pab1_ and proteins bound—including eIF4G1, Pab1, Sbp1, and its domain variants—were mixed at different stoichiometric ratios. Binding of the RNA to the protein was examined by electrophoresis in an 8% (bis-acrylamide 29:1) polyacrylamide under native conditions in THEM buffer (34 mM Tris Base, 57 mM Hepes, 0.1 mM EDTA, 2.5 mM MgCl_2_). Bound and unbound RNAs were quantified using ImageQuant software (Molecular Dynamics). The dissociation constant (*K*_d_) and the Hill coefficient (*n*) was obtained by fitting data to the following equation:

$$\frac{[\text{RNA}{]}_{\mathrm{bound}}}{\left([\text{RNA}{]}_{\mathrm{bound}}+[\text{RNA}{]}_{\mathrm{free}}\right)}=\frac{[\text{P}{]}_{\mathrm{total}}^{n}}{\left(K_{d}^{n}+[\text{P}{]}_{\mathrm{total}}^{n}\right)},$$

where [P]_total_ is the total concentration of the protein, [P]_total_ ≈ [P]_free_ under the conditions used, and [RNA]_bound_ and [RNA]_free_ are concentrations of RNA in the protein-bound and free forms, respectively.

### GST pulldowns

GST-tagged Pab1 or Sbp1, immobilized on a glutathione–sepharose resin, was incubated with the target proteins or their mutants containing individual domains with or without 5′UTR_Pab1_ at 4°C for 30 min in the binding buffer (50 mM Tris–HCl, pH 7.5, 120 mM KCl, 2 mM DTT, 2 mM MgCl_2_, 0.5% Triton-X-100). The resin was then washed four times with cold binding buffer and subsequently boiled in 1× SDS-loading buffer (50 mM Tris–HCl pH 6.8, 2% SDS, 10% Glycerol, 1% β-mercaptoethanol, 0.02% bromophenol blue) to elute bound proteins. RNase treatment was carried out after the formation of RNP by incubating RNase A with the RNP at 25°C for 30 min. The proteins were resolved by SDS–PAGE and detected by either Coomassie Brilliant Blue or SYPRO Ruby staining.

### In vivo methylation

Methylation of the arginine in the RGG domain of Sbp1 was done essentially as previously described (Hsieh et al. 2007) with the following modifications. Briefly, plasmids containing Sbp1 and type I arginine methyltransferase Hmt1 were cotransformed and coexpressed in *E. coli* BL21 (DE3) cells. Methylated Sbp1 was purified by affinity chromatography followed by ion-exchange chromatography using HiTrap Heparin HP column (GE Healthcare). Methylation of the arginines in the RGG domain was confirmed by LC–MS/MS.

### Western blot and northern blot

Western blot was done as previously described (Coller and Parker 2005; Sweet et al. 2012; Zeng and Jin 2016). Briefly, fractions from polysome profiling were resolved by SDS–PAGE; and proteins in unfixed gels were transferred to nitrocellulose membranes (GenScript) for 30 min at 100 V in a Mini Trans-Blot apparatus (Bio-Rad). Protein bands were detected with anti-GST (Invitrogen) or anti-His antibodies (Sigma-Aldrich). Northern blots were done as previously described (Coller and Parker 2005; Sweet et al. 2012). RNA samples were separated on a 5% urea acrylamide gel, transferred to nylon membranes, and probed overnight with a luciferase gene-specific ^32^P-labeled DNA probe. Blots were exposed to PhosphorImager screens, scanned by a Storm 840 scanner, and quantified with ImageQuant software.

## SUPPLEMENTAL MATERIAL

Supplemental material is available for this article.

## Supplementary Material

Supplemental Material
