# Supplementary material for: Sbp1 modulates the translation of Pab1 mRNA in a poly(A)- and RGG-dependent manner
Source: RNA. 2018 Jan;24(1):43–55. doi: 10.1261/rna.062547.117 (PMC5733569; doi:10.1261/rna.062547.117)
Supplement: Supplemental Material [file supp_24_1_43__index.html]

Sbp1 modulates the translation of Pab1 mRNA in a poly(A)- and RGG-dependent manner — Supplemental Material 

# Sbp1 modulates the translation of Pab1 mRNA in a poly(A)- and RGG-dependent manner

## Supplemental Material

- Supplemental\_Figure\_S1.tif
- Supplemental\_Figure\_S2.tif
- Supplemental\_Figure\_S3.tif
- Supplemental\_Figure\_S4.tif
- Supplemental\_Figure\_S5.tif
- Supplemental\_Figure\_S6.tif
- Supplemental\_Figure\_S7.tif
- Supplemental\_Reference.docx
