# Supplementary figures and images for: Sbp1 modulates the translation of Pab1 mRNA in a poly(A)- and RGG-dependent manner
Source: RNA. 2018 Jan;24(1):43–55. doi: 10.1261/rna.062547.117 (PMC5733569; doi:10.1261/rna.062547.117)

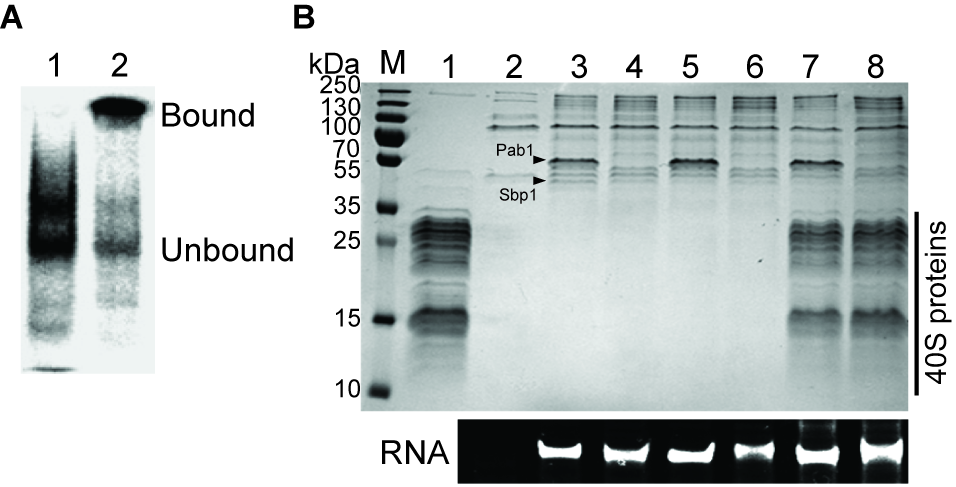

Supplement: Supplemental Material [file supp_062547.117_Supplemental_Figure_S1.tif]

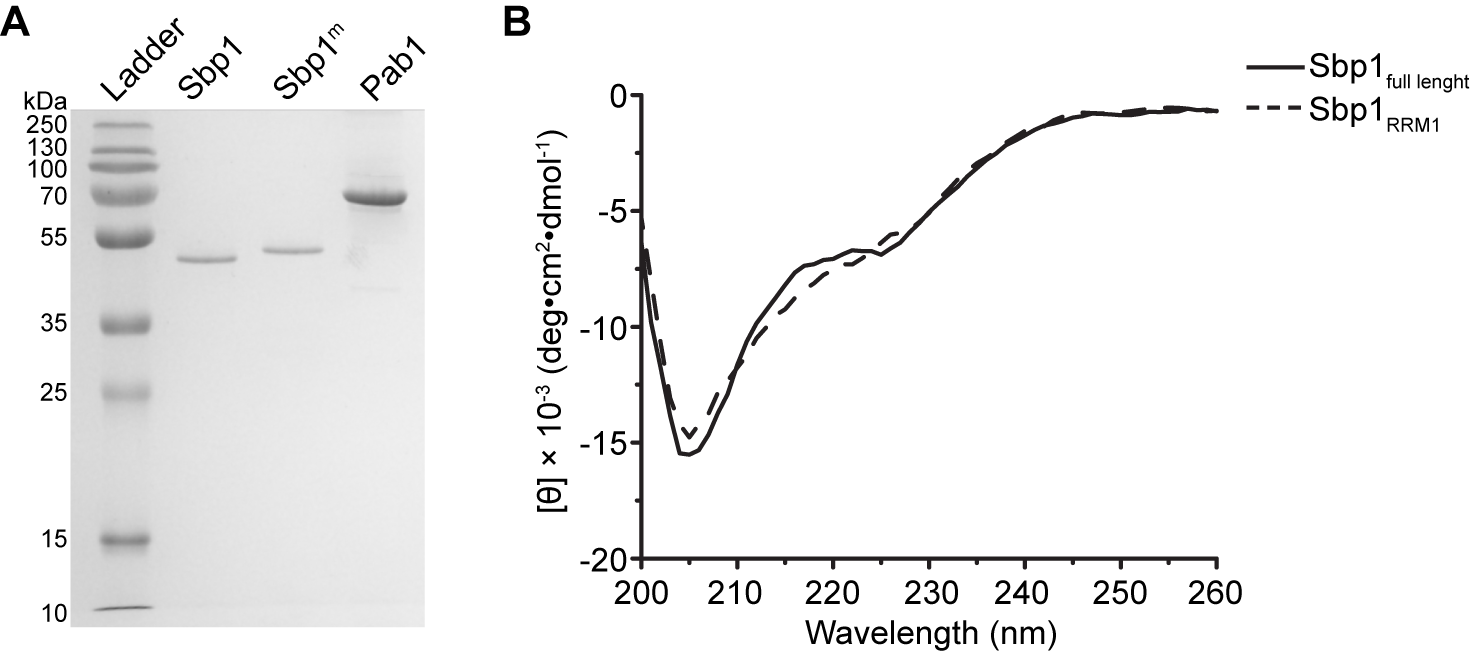

Supplement: Supplemental Material [file supp_062547.117_Supplemental_Figure_S2.tif]

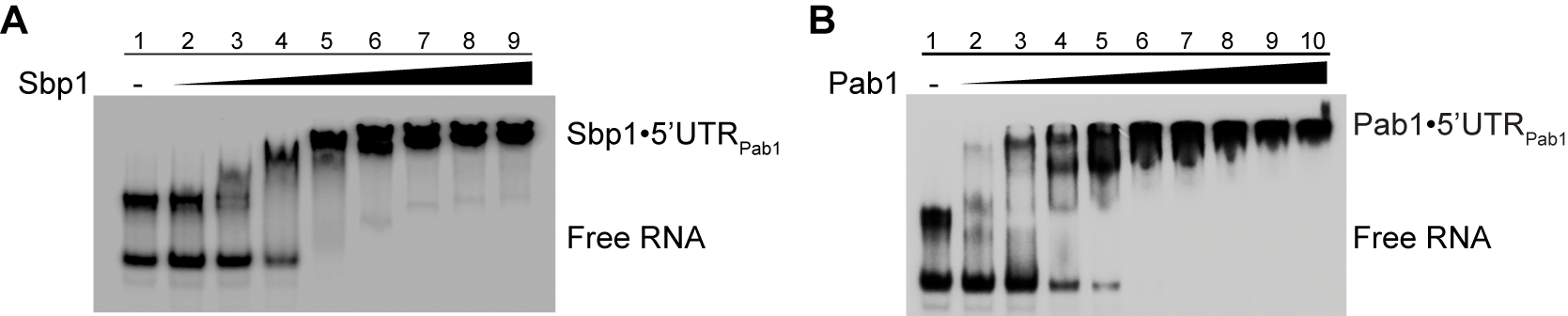

Supplement: Supplemental Material [file supp_062547.117_Supplemental_Figure_S3.tif]

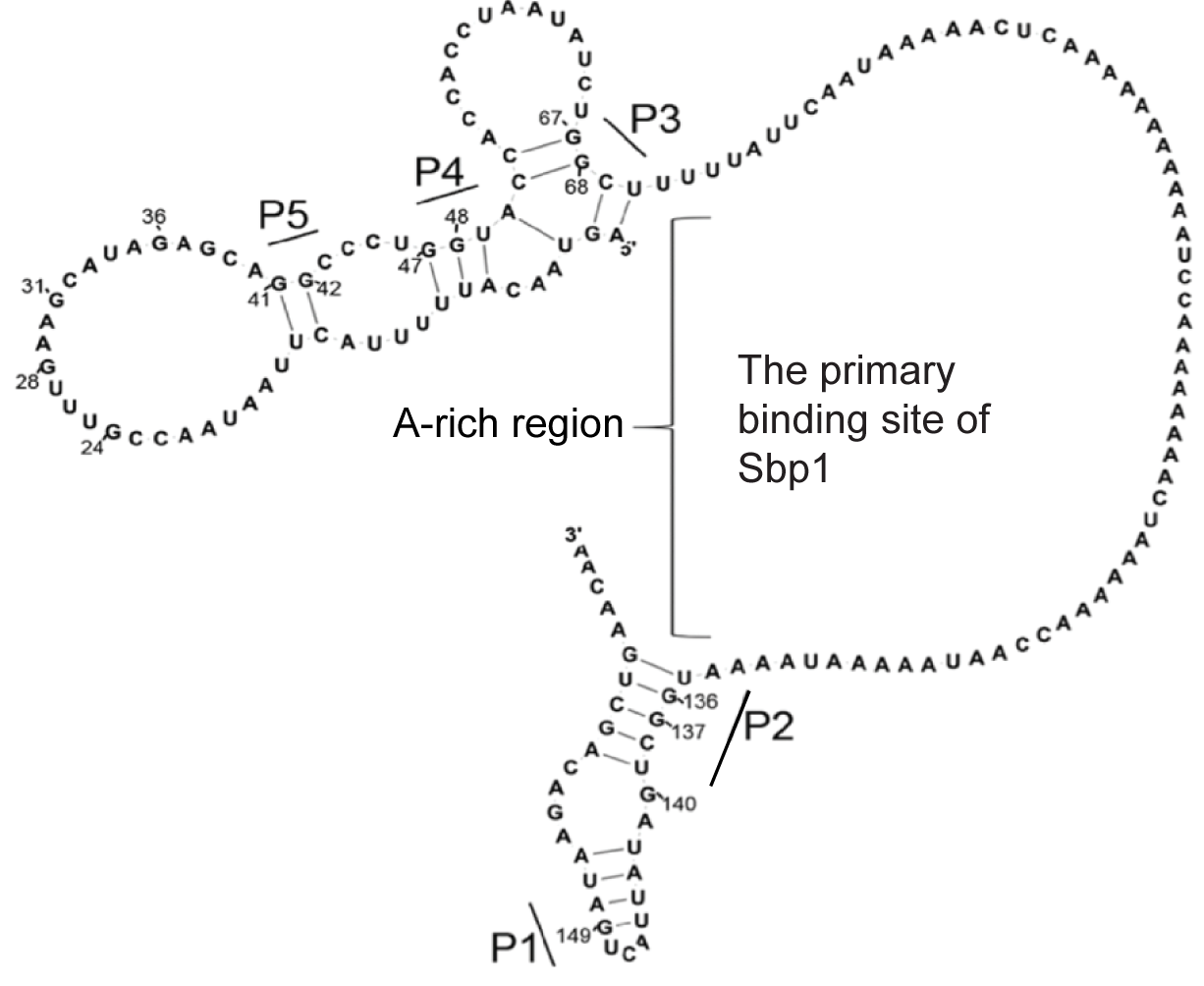

Supplement: Supplemental Material [file supp_062547.117_Supplemental_Figure_S4.tif]

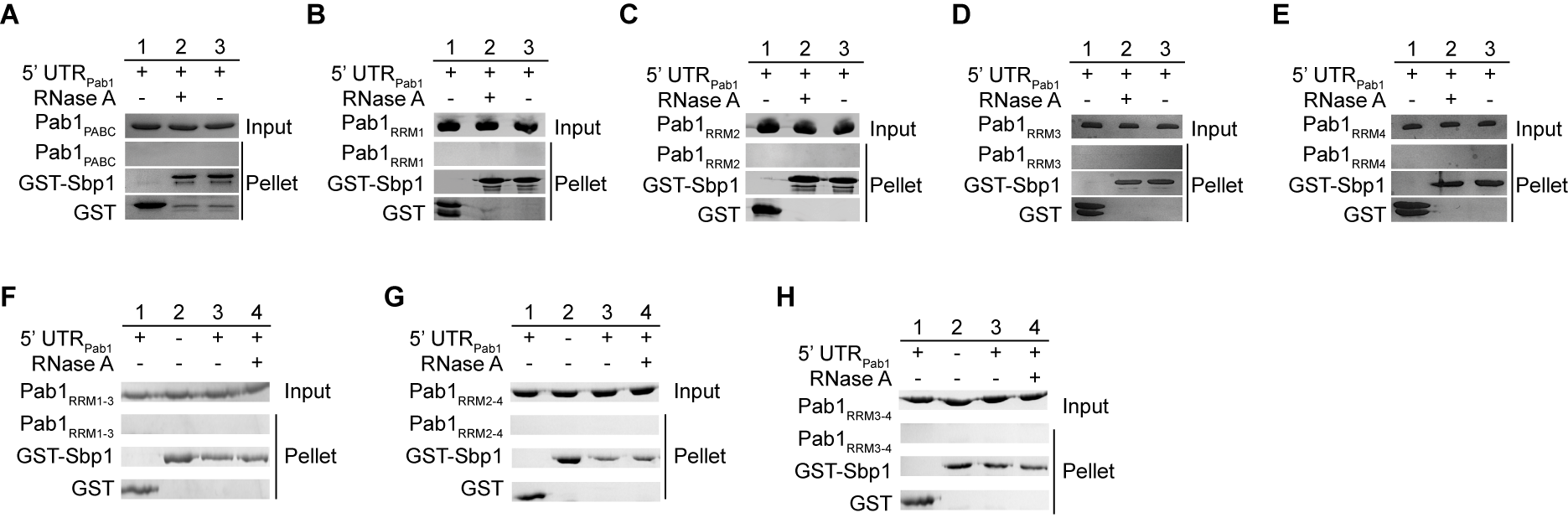

Supplement: Supplemental Material [file supp_062547.117_Supplemental_Figure_S5.tif]

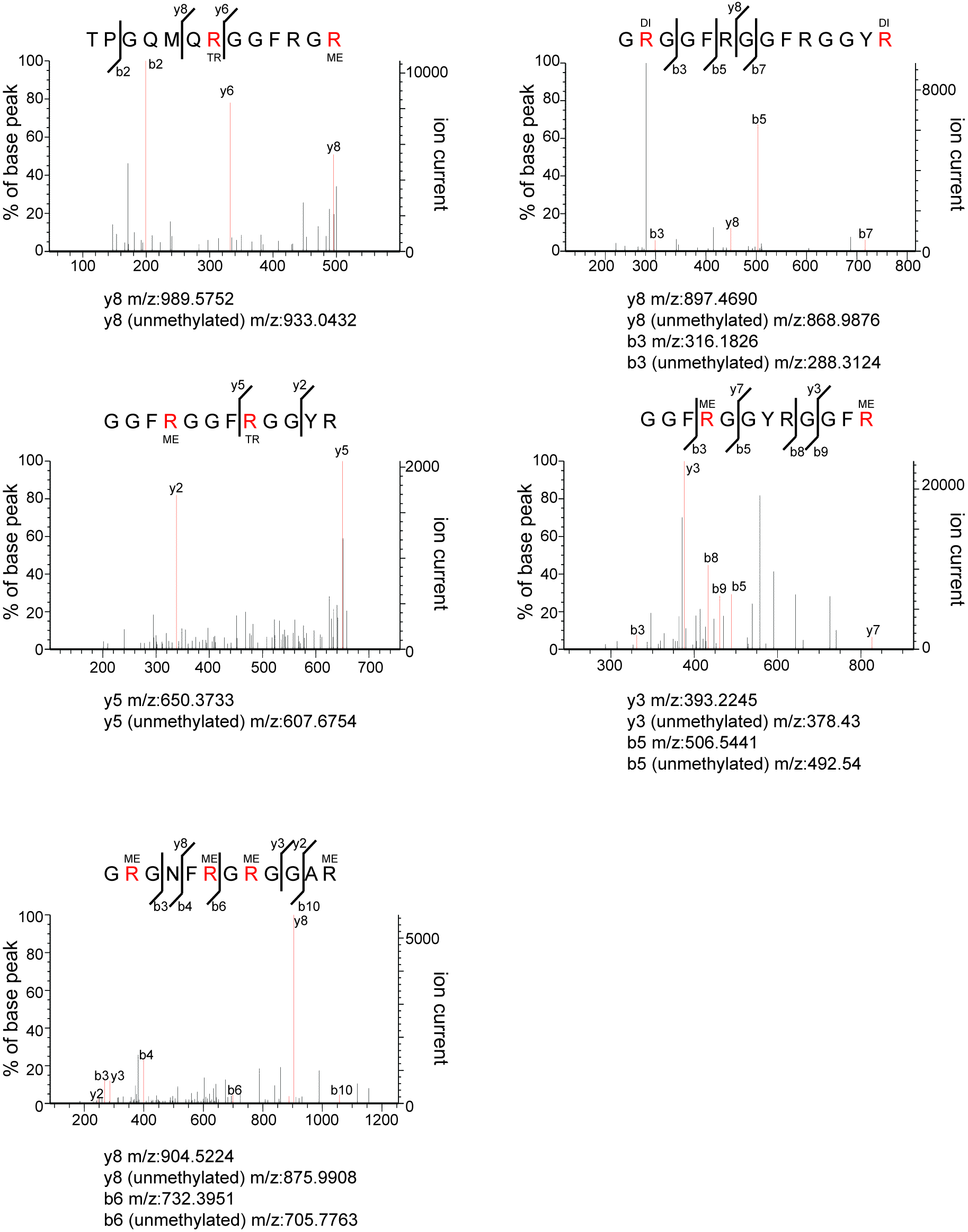

Supplement: Supplemental Material [file supp_062547.117_Supplemental_Figure_S6.tif]

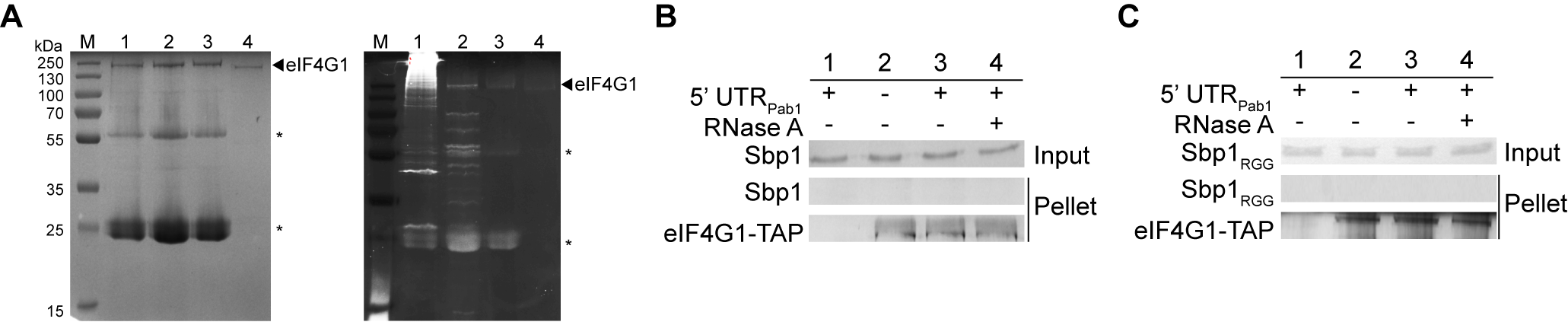

Supplement: Supplemental Material [file supp_062547.117_Supplemental_Figure_S7.tif]
