# Supplementary material for: Sbp1 modulates the translation of Pab1 mRNA in a poly(A)- and RGG-dependent manner
Source: RNA. 2018 Jan;24(1):43–55. doi: 10.1261/rna.062547.117 (PMC5733569; doi:10.1261/rna.062547.117)
Supplement: Supplemental Material [file supp_062547.117_Supplemental_Reference.docx]

**SUPPLEMENTAL REFERENCE**

Zuker M. 2003. Mfold web server for nucleic acid folding and hybridization prediction. *Nucleic Acids Res* **31**: 3406-3415.
